# Crystal structure of (±)-(5*SR*,6*SR*)-6-ethenyl-1-[(*RS*)-1-phenyl­eth­oxy]-1-aza­spiro­[4.5]decan-2-one

**DOI:** 10.1107/S2056989015021209

**Published:** 2015-11-25

**Authors:** Takeshi Oishi, Shio Yamamoto, Takashi Yokoyama, Akihiro Kobayashi, Takaaki Sato, Noritaka Chida

**Affiliations:** a School of Medicine, Keio University, Hiyoshi 4-1-1, Kohoku-ku, Yokohama 223-8521, Japan; b Department of Applied Chemistry, Faculty of Science and Technology, Keio University, Hiyoshi 3-14-1, Kohoku-ku, Yokohama 223-8522, Japan

**Keywords:** crystal structure, pyrrolidine, cyclo­hexa­ne, *N*-alk­oxy-*N*-alkyl­amide, hydrogen bonding

## Abstract

In the title compound, C_19_H_25_NO_2_, the pyrrolidine ring adopts an envelope form, with the spiro C atom as the flap, while the cyclo­hexane ring shows a chair form. A weak intra­molecular C—H⋯O inter­action supports the mol­ecular conformation, generating an *S*(6) ring motif. In the crystal, pairs of C—H⋯O inter­actions connect the mol­ecules into inversion dimers with an *R*_2_^2^(16) ring motif. The dimers are linked by a second pair of C—H⋯O inter­actions, enclosing an *R*_4_^2^(12) ring motif, into a tape structure along the *b* axis.

## Abstract 2

In the title compound, the pyrrolidine and cyclo­hexane rings exhibit envelope and chair conformations, respectively. In the crystal, C—H⋯O inter­actions connect the mol­ecules into tape structures.

## Chemical context   

A number of compounds containing an *N*-hy­droxy or *N*-alk­oxy substituent have been widely explored in organic synthesis. These substances show specific and intriguing reactivity caused by a covalent bond between the electronegative heteroatoms. Among these compounds, for example, the *N*-alk­oxy­amines are known to be initiators for stable free radical polymerization (Hawker *et al.*, 2001 ▸), and the *N*-alk­oxy­amides are utilized for mild and effective acyl­ating agents (*cf.* Weinreb amide; Nahm & Weinreb, 1981 ▸). We noticed this stable but contributable functionality, and have developed a new synthetic pathway to synthesize the natural alkaloids (Sato & Chida, 2014 ▸).
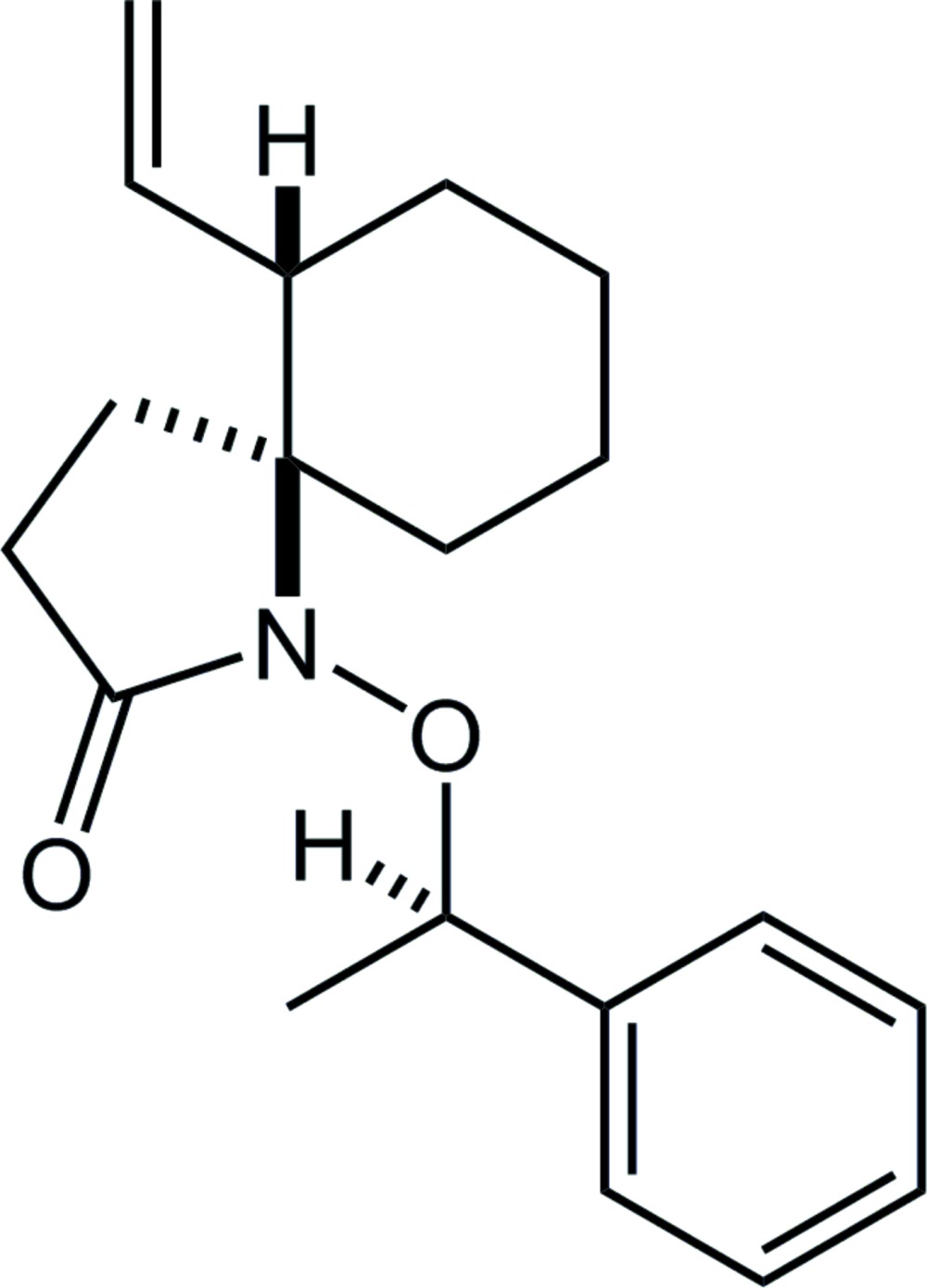


## Structural commentary   

**Figure 1:**
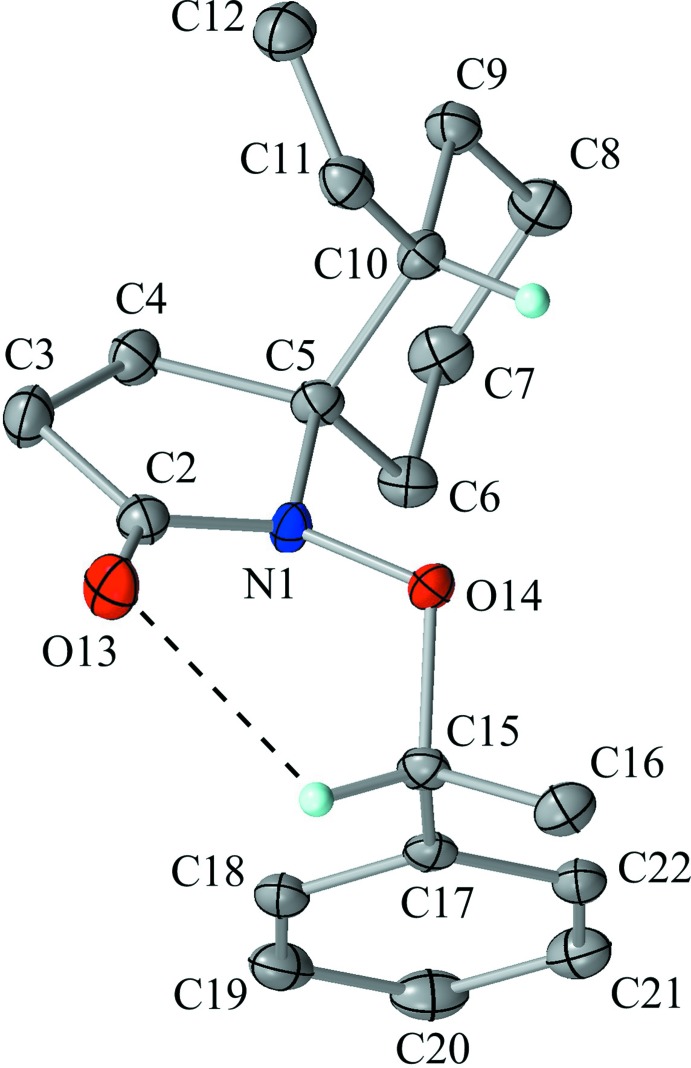
The mol­ecular structure of the title compound, showing the the atom labelling. Displacement ellipsoids are drawn at the 50% probability level. The black dashed line indicates the intra­molecular C—H⋯O hydrogen bond. Only H atoms connected to chiral C atoms are shown for clarity.

The mol­ecular structure of the title compound is shown in Fig. 1 ▸. The pyrrolidine ring (N1/C2–C5) adopts an envelope form, with puckering parameters of *Q*(2) = 0.1965 (16) Å and *φ*(2) = 151.8 (5)°. The flap atom C5 deviates from the mean plane of other four atoms by 0.314 (2) Å. For the *N*-alk­oxy-*N*-alkyl­amide moiety, the geometry around atom N1 is a little deformed from a planar to a pyramidal configuration. The shift of atom N1 from the C2/C5/O14 plane is 0.2163 (13) Å, and the sum of angles for C2—N1—O14, O14—N1—C5 and C5—N1—C2 is 353.0°.

The cyclo­hexane ring (C5–C10), which is spiro-fused to the pyrrolidine ring, adopts a chair form with puckering parameters of *Q* = 0.5782 (17) Å, *θ* = 1.82 (17)°, *φ* = 347 (5)°, *Q*(2) = 0.0197 (17) Å and *Q*(3) = 0.5779 (17) Å. The equatorially oriented C10—C11 bond makes an angle of 70.60 (9)° with the normal to the Cremer & Pople plane of the cyclo­hexane ring, and the vinyl group (C11=C12) is positioned in syn-periplanar geometry to the cyclo­hexane framework, with a C9—C10—C11=C12 torsion angle of 10.9 (2)°.

An intra­molecular C—H⋯O inter­action (C15—H15⋯O13) supports the mol­ecular conformation, generating an *S*(6) graph-set motif. No intra­molecular C—H⋯π inter­action is observed.

## Supra­molecular features   

**Figure 2:**
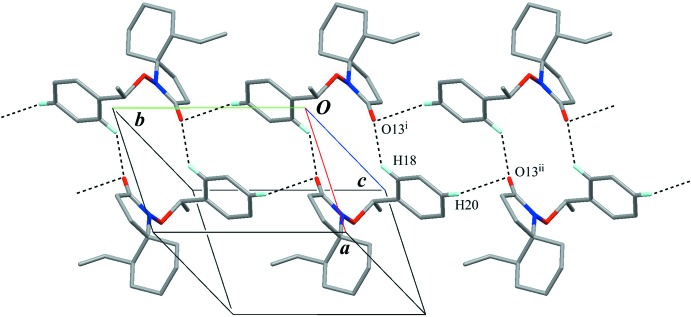
A partial packing view showing the tape structure. Black dashed lines indicate the inter­molecular C—H⋯O hydrogen bonds. Only H atoms involved in the hydrogen bonds are shown for clarity. [Symmetry codes: (i) −*x*, −*y*, −*z* + 1; (ii) *x*, *y* − 1, *z*.]

In the crystal, a pair of C—H⋯O inter­actions (C18—H18⋯O13^i^; Table 1 ▸) with an 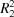
(16) graph-set motif links the mol­ecules, forming an inversion dimer. The dimers are linked into a tape structure running along the *b* axis by weak C—H⋯O inter­actions (C20—H20⋯O13^ii^; Table 1 ▸), enclosing an 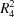
(12) graph-set motif (Figs. 2 ▸ and 3 ▸). There is no inter­molecular C—H⋯π inter­action.

## Database survey   

**Figure 4:**
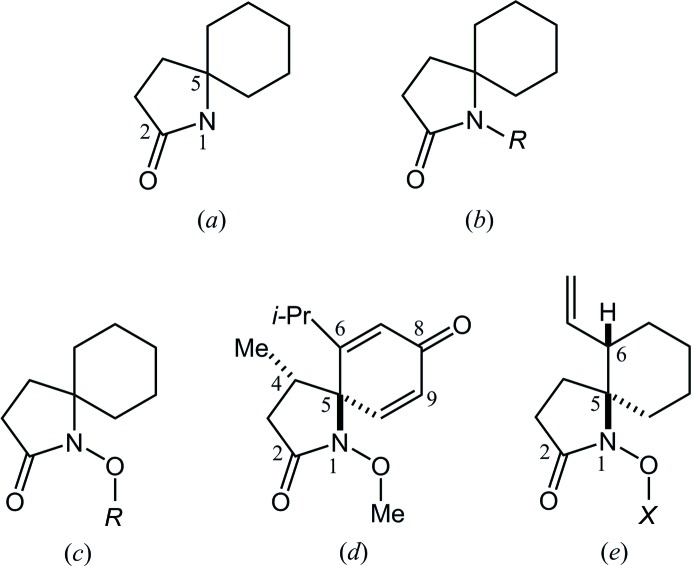
The structures of (*a*) the 1-aza­spiro­[4.5]decan-2-one skeleton from the database survey and its (*b*) *N*-alkyl and (*c*) *N*-alk­oxy derivatives; *R* = alkyl or aryl, (*d*) (4S*,5R*)-6-isopropyl-1-meth­oxy-4-methyl-1-aza­spiro­[4.5]deca-6,9-diene-2,8-dione and (*e*) the title compound; *X* = (R*)-1-phenyl­ethyl.

In the Cambridge Structural Database (CSD, Version 5.36, November 2014; Groom & Allen, 2014 ▸), 20 structures containing a 1-aza­spiro­[4.5]decan-2-one skeleton, (*a*), are registered (Fig. 4 ▸). These include 14 compounds with an *N*-alkyl substituent, (*b*), but no compound with an *N*-alk­oxy substituent, (*c*).

The structure of an *N*-meth­oxy-aza­spiro­cyclic derivative, (*d*), which is related to the title compound, (*e*), has also been reported (TUWCUJ; Wardrop *et al.*, 2003 ▸). In the crystal of (*d*), the pyrrolidine ring adopts a similar conformation to the title compound. The spiro-C atom is at the flap of the envelope, and the geometry around the N atom shows a little deformation to a pyramidal configuration with the sum of the C(carbon­yl)—N—O, O—N—C and C—N—C(carbon­yl) angles being 345.8 (5)°. No intra­molecular C—H⋯O inter­action is observed in (*d*).

## Synthesis and crystallization   

The title compound was synthesized convergently from hex-5-en-1-ol, methyl 4-chloro-4-oxobutyrate and 1-phenyl­ethanol (Yamamoto *et al.*, 2015 ▸). Purification was carried out by silica gel column chromatography, and colorless crystals were obtained from a hexane solution by slow evaporation at ambient temperature. M.p. 357.1–357.8 K. HRMS (ESI) *m*/*z* calculated for C_19_H_25_NO_2_Na^+^ [*M* + Na]^+^: 322.1783; found: 322.1779. Analysis calculated for C_19_H_25_NO_2_: C 76.22, H 8.42, N 4.68%; found: C 76.31, H 8.44, N 4.58%.

## Refinement   

Crystal data, data collection and structure refinement details are summarized in Table 2 ▸. C-bound H atoms were positioned geometrically with C—H = 0.95–1.00 Å, and constrained to ride on their parent atoms with *U*_iso_(H) = 1.2*U*_eq_(C) or 1.5*U*_eq_(methyl C).

## Supplementary Material

Crystal structure: contains datablock(s) global, I. DOI: 10.1107/S2056989015021209/is5431sup1.cif

Structure factors: contains datablock(s) I. DOI: 10.1107/S2056989015021209/is5431Isup2.hkl

Supporting information file. DOI: 10.1107/S2056989015021209/is5431Isup3.cml

CCDC reference: 1435676

Additional supporting information:  crystallographic information; 3D view; checkCIF report

## Figures and Tables

**Figure 3:**
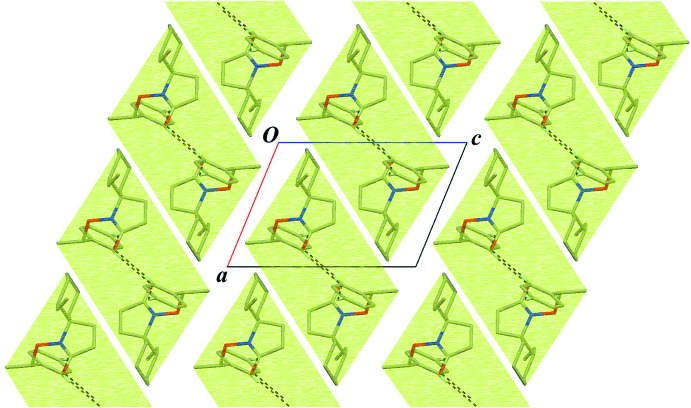
A packing diagram viewed down the *b* axis. Black dotted lines indicate the inter­molecular C—H⋯O inter­actions. The pale-green parallelograms indicate the tape structures running along the *b* axis. Only H atoms involved in hydrogen bonding are shown for clarity.

**Table 1:** Hydrogen-bond geometry (Å, °)

*D*—H⋯*A*	*D*—H	H⋯*A*	*D*⋯*A*	*D*—H⋯*A*
C15—H15⋯O13	1.00	2.42	3.0437 (16)	120
C18—H18⋯O13^i^	0.95	2.53	3.2864 (17)	136
C20—H20⋯O13^ii^	0.95	2.61	3.4307 (17)	145

Symmetry codes: (i) 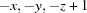; (ii) 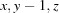.

**Table 2:** Experimental details

Crystal data	
Chemical formula	C_19_H_25_NO_2_
*M*_r_	299.41
Crystal system, space group	Triclinic, *P* 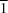
Temperature (K)	90
*a*, *b*, *c* (Å)	8.9032 (5), 9.6307 (5), 11.3401 (6)
α, β, γ (°)	93.306 (2), 108.710 (2), 114.929 (2)
*V* (Å^3^)	813.83 (8)
*Z*	2
Radiation type	Mo *K*α
μ (mm^−1^)	0.08
Crystal size (mm)	0.29 × 0.24 × 0.20

Data collection	
Diffractometer	Bruker D8 Venture
Absorption correction	Multi-scan (*SADABS*; Bruker, 2014 ▸)
*T*_min_, *T*_max_	0.98, 0.98
No. of measured, independent and observed [*I* > 2σ(*I*)] reflections	14217, 2861, 2304
*R*_int_	0.028
(sin θ/λ)_max_ (Å^−1^)	0.595

Refinement	
*R*[*F* ^2^ > 2σ(*F* ^2^)], *wR*(*F* ^2^), *S*	0.034, 0.083, 1.06
No. of reflections	2861
No. of parameters	200
H-atom treatment	H-atom parameters constrained
Δρ_max_, Δρ_min_ (e Å^−3^)	0.23, −0.21

Computer programs: *APEX2* and *SAINT* (Bruker, 2014 ▸), *SHELXS2013* (Sheldrick, 2008 ▸), *SHELXL2014* (Sheldrick, 2015 ▸), *Mercury* (Macrae *et al.*, 2006 ▸), *publCIF* (Westrip, 2010 ▸) and *PLATON* (Spek, 2009 ▸).

## supplementary crystallographic information

### Crystal data

**Table:**

C_19_H_25_NO_2_	*F*(000) = 324
*M**_r_* = 299.41	*D*_x_ = 1.222 Mg m^−^^3^
Triclinic, *P*1	Melting point: 357.8 K
*a* = 8.9032 (5) Å	Mo *K*α radiation, λ = 0.71073 Å
*b* = 9.6307 (5) Å	Cell parameters from 5086 reflections
*c* = 11.3401 (6) Å	θ = 2.7–25.1°
α = 93.306 (2)°	µ = 0.08 mm^−^^1^
β = 108.710 (2)°	*T* = 90 K
γ = 114.929 (2)°	Plate, colorless
*V* = 813.83 (8) Å^3^	0.29 × 0.24 × 0.20 mm
*Z* = 2	

### Data collection

**Table:**

Bruker D8 Venture diffractometer	2861 independent reflections
Radiation source: fine-focus sealed tube	2304 reflections with *I* > 2σ(*I*)
Multilayered confocal mirror monochromator	*R*_int_ = 0.028
Detector resolution: 10.4167 pixels mm^-1^	θ_max_ = 25.0°, θ_min_ = 2.4°
φ and ω scans	*h* = −10→10
Absorption correction: multi-scan (*SADABS*; Bruker, 2014)	*k* = −11→11
*T*_min_ = 0.98, *T*_max_ = 0.98	*l* = −13→13
14217 measured reflections	

### Refinement

**Table:**

Refinement on *F*^2^	Primary atom site location: structure-invariant direct methods
Least-squares matrix: full	Secondary atom site location: difference Fourier map
*R*[*F*^2^ > 2σ(*F*^2^)] = 0.034	Hydrogen site location: inferred from neighbouring sites
*wR*(*F*^2^) = 0.083	H-atom parameters constrained
*S* = 1.06	*w* = 1/[σ^2^(*F*_o_^2^) + (0.0345*P*)^2^ + 0.2546*P*] where *P* = (*F*_o_^2^ + 2*F*_c_^2^)/3
2861 reflections	(Δ/σ)_max_ = 0.003
200 parameters	Δρ_max_ = 0.23 e Å^−^^3^
0 restraints	Δρ_min_ = −0.21 e Å^−^^3^

### Special details

**Table:**

Experimental. IR (film): 2933, 2859, 1708, 1451, 1047, 916, 700 cm^-1^; ^1^H NMR (500 MHz, CDCl_3_): *δ* (p.p.m.) 7.41–7.38 (m, 2H; H18 & H22), 7.37–7.28 (m, 3H; H19–21), 5.66 (ddd, *J* = 17.5, 10.6, 6.9 Hz, 1H; H11), 5.27 (q, *J* = 6.6 Hz, 1H; H15), 5.15 (ddd, *J* = 17.5, 1.7, 1.4 Hz, 1H; H12A), 5.09 (ddd, *J* = 10.6, 1.7, 1.2 Hz, 1H; H12B), 2.46 (ddddd, *J* = 11.5, 6.9, 4.0, 1.4, 1.2 Hz, 1H; H10), 2.24 (ddd, *J* = 17.5, 10.9, 3.7 Hz, 1H; H3B), 2.17 (ddd, *J* = 17.5, 10.3, 7.8 Hz, 1H; H3A), 1.98 (ddd, *J* = 14.0, 10.3, 3.7 Hz, 1H; H4A), 1.72–1.68 (m, 1H; H9B), 1.66 (d, *J* = 6.6 Hz, 3H; H16ABC), 1.63–1.57 (m, 1H; H7B), 1.49–1.41 (m, 2H; H4B & H8B), 1.31–1.20 (m, 2H; H6A & H9A), 1.17–1.01 (m, 2H; H7A & H8A), 0.90–0.82 (m, 1H; H6B). ^13^C NMR (125 MHz, CDCl_3_): *δ* (p.p.m.) 172.8 (C; C2), 141.5 (C; C17), 137.6 (CH; C11), 128.4 (CH; C20), 128.3 (CH; C19 & C21), 127.7 (CH; C18 & C22), 117.8 (CH_2_; C12), 83.2 (CH; C15), 66.7 (C; C5), 44.6 (CH; C10), 37.6 (CH_2_; C6), 27.7 (CH_2_; C9), 27.3 (CH_2_; C3), 25.0 (CH_2_; C7), 22.8 (CH_2_; C8), 22.6 (CH_2_; C4), 21.2 (CH_3_; C16).
Geometry. All e.s.d.'s (except the e.s.d. in the dihedral angle between two l.s. planes) are estimated using the full covariance matrix. The cell e.s.d.'s are taken into account individually in the estimation of e.s.d.'s in distances, angles and torsion angles; correlations between e.s.d.'s in cell parameters are only used when they are defined by crystal symmetry. An approximate (isotropic) treatment of cell e.s.d.'s is used for estimating e.s.d.'s involving l.s. planes.
Refinement. Refinement of *F*^2^ against ALL reflections. The weighted *R*-factor *wR* and goodness of fit *S* are based on *F*^2^, conventional *R*-factors *R* are based on *F*, with *F* set to zero for negative *F*^2^. The threshold expression of *F*^2^ > 2*σ*(*F*^2^) is used only for calculating *R*-factors(gt) *etc.* and is not relevant to the choice of reflections for refinement. *R*-factors based on *F*^2^ are statistically about twice as large as those based on *F*, and *R*-factors based on ALL data will be even larger.Problematic one reflection with |*I*(obs)-*I*(calc)|/*σ*W(*I*) greater than 10 (5 4 0) has been omitted in the final refinement.

### Fractional atomic coordinates and isotropic or equivalent isotropic displacement parameters (Å^2^)

**Table:**

	*x*	*y*	*z*	*U*_iso_*/*U*_eq_	
N1	0.38594 (14)	0.18774 (12)	0.71854 (10)	0.0150 (3)	
C2	0.27948 (18)	0.23274 (15)	0.63314 (13)	0.0181 (3)	
C3	0.35545 (19)	0.27324 (18)	0.53172 (13)	0.0239 (3)	
H3A	0.3831	0.3823	0.5236	0.029*	
H3B	0.2694	0.2009	0.448	0.029*	
C4	0.52667 (18)	0.25541 (17)	0.57599 (12)	0.0204 (3)	
H4A	0.6283	0.3505	0.5734	0.024*	
H4B	0.5107	0.1636	0.5197	0.024*	
C5	0.56353 (17)	0.23207 (15)	0.71449 (12)	0.0161 (3)	
C6	0.61336 (18)	0.09955 (16)	0.73662 (13)	0.0187 (3)	
H6A	0.6112	0.0754	0.8199	0.022*	
H6B	0.5232	0.0039	0.6691	0.022*	
C7	0.79862 (18)	0.14278 (17)	0.73554 (14)	0.0224 (3)	
H7A	0.7978	0.157	0.6496	0.027*	
H7B	0.8281	0.056	0.7539	0.027*	
C8	0.94114 (18)	0.29350 (17)	0.83460 (14)	0.0239 (3)	
H8A	0.9525	0.2752	0.9213	0.029*	
H8B	1.0582	0.3232	0.8274	0.029*	
C9	0.89331 (18)	0.42710 (16)	0.81564 (13)	0.0207 (3)	
H9A	0.8956	0.4533	0.7329	0.025*	
H9B	0.9842	0.5214	0.8842	0.025*	
C10	0.70790 (17)	0.38388 (15)	0.81750 (13)	0.0169 (3)	
H10	0.7115	0.358	0.9022	0.02*	
C11	0.65709 (18)	0.51421 (16)	0.80790 (12)	0.0197 (3)	
H11	0.5532	0.4978	0.824	0.024*	
C12	0.7417 (2)	0.64903 (16)	0.77944 (13)	0.0250 (3)	
H12A	0.8464	0.6713	0.7625	0.03*	
H12B	0.6981	0.7239	0.7759	0.03*	
O13	0.14599 (13)	0.23609 (11)	0.63826 (9)	0.0236 (2)	
O14	0.36946 (11)	0.17339 (10)	0.83675 (8)	0.0157 (2)	
C15	0.20748 (17)	0.02915 (15)	0.82165 (12)	0.0162 (3)	
H15	0.1019	0.0301	0.7556	0.019*	
C16	0.19176 (19)	0.04574 (16)	0.94965 (13)	0.0205 (3)	
H16A	0.3004	0.0563	1.0167	0.031*	
H16B	0.0876	−0.0475	0.949	0.031*	
H16C	0.1771	0.1393	0.9664	0.031*	
C17	0.22033 (17)	−0.11480 (15)	0.77798 (12)	0.0161 (3)	
C18	0.14415 (17)	−0.18306 (16)	0.64821 (13)	0.0193 (3)	
H18	0.0813	−0.1409	0.5897	0.023*	
C19	0.15846 (19)	−0.31154 (16)	0.60300 (14)	0.0236 (3)	
H19	0.1063	−0.3567	0.514	0.028*	
C20	0.24866 (19)	−0.37425 (16)	0.68731 (14)	0.0257 (3)	
H20	0.2588	−0.4624	0.6564	0.031*	
C21	0.32436 (19)	−0.30816 (16)	0.81723 (14)	0.0228 (3)	
H21	0.3859	−0.3515	0.8755	0.027*	
C22	0.31044 (17)	−0.17913 (15)	0.86219 (13)	0.0178 (3)	
H22	0.3628	−0.1342	0.9513	0.021*	

### Atomic displacement parameters (Å^2^)

**Table:**

	*U*^11^	*U*^22^	*U*^33^	*U*^12^	*U*^13^	*U*^23^
N1	0.0159 (6)	0.0195 (6)	0.0124 (6)	0.0089 (5)	0.0073 (4)	0.0061 (5)
C2	0.0188 (7)	0.0155 (7)	0.0185 (7)	0.0079 (6)	0.0056 (6)	0.0034 (6)
C3	0.0249 (8)	0.0299 (8)	0.0180 (7)	0.0131 (7)	0.0086 (6)	0.0095 (6)
C4	0.0212 (7)	0.0236 (8)	0.0171 (7)	0.0096 (6)	0.0093 (6)	0.0045 (6)
C5	0.0139 (7)	0.0186 (7)	0.0176 (7)	0.0070 (6)	0.0087 (6)	0.0044 (6)
C6	0.0176 (7)	0.0172 (7)	0.0221 (7)	0.0081 (6)	0.0089 (6)	0.0033 (6)
C7	0.0213 (8)	0.0246 (8)	0.0281 (8)	0.0137 (7)	0.0133 (6)	0.0073 (6)
C8	0.0169 (7)	0.0295 (8)	0.0286 (8)	0.0117 (7)	0.0111 (6)	0.0086 (7)
C9	0.0169 (7)	0.0222 (8)	0.0204 (7)	0.0065 (6)	0.0075 (6)	0.0043 (6)
C10	0.0176 (7)	0.0178 (7)	0.0160 (7)	0.0077 (6)	0.0078 (6)	0.0050 (6)
C11	0.0187 (7)	0.0203 (8)	0.0187 (7)	0.0082 (6)	0.0071 (6)	0.0019 (6)
C12	0.0284 (8)	0.0212 (8)	0.0251 (8)	0.0113 (7)	0.0103 (6)	0.0050 (6)
O13	0.0204 (5)	0.0311 (6)	0.0255 (5)	0.0165 (5)	0.0092 (4)	0.0106 (5)
O14	0.0160 (5)	0.0175 (5)	0.0138 (5)	0.0060 (4)	0.0080 (4)	0.0046 (4)
C15	0.0112 (7)	0.0171 (7)	0.0199 (7)	0.0053 (6)	0.0070 (5)	0.0055 (6)
C16	0.0215 (7)	0.0217 (7)	0.0244 (8)	0.0116 (6)	0.0136 (6)	0.0074 (6)
C17	0.0107 (6)	0.0164 (7)	0.0199 (7)	0.0034 (6)	0.0082 (6)	0.0058 (6)
C18	0.0133 (7)	0.0198 (7)	0.0199 (7)	0.0034 (6)	0.0062 (6)	0.0050 (6)
C19	0.0217 (8)	0.0185 (7)	0.0229 (8)	0.0018 (6)	0.0105 (6)	−0.0006 (6)
C20	0.0262 (8)	0.0161 (7)	0.0362 (9)	0.0073 (7)	0.0177 (7)	0.0024 (7)
C21	0.0203 (7)	0.0195 (8)	0.0322 (9)	0.0100 (6)	0.0125 (6)	0.0098 (6)
C22	0.0145 (7)	0.0180 (7)	0.0192 (7)	0.0051 (6)	0.0076 (6)	0.0050 (6)

### Geometric parameters (Å, º)

**Table:**

N1—C2	1.3528 (17)	C10—C11	1.5015 (18)
N1—O14	1.4028 (13)	C10—H10	1.0
N1—C5	1.4712 (16)	C11—C12	1.3182 (19)
C2—O13	1.2220 (16)	C11—H11	0.95
C2—C3	1.5035 (19)	C12—H12A	0.95
C3—C4	1.5313 (19)	C12—H12B	0.95
C3—H3A	0.99	O14—C15	1.4711 (15)
C3—H3B	0.99	C15—C17	1.5070 (18)
C4—C5	1.5483 (18)	C15—C16	1.5082 (18)
C4—H4A	0.99	C15—H15	1.0
C4—H4B	0.99	C16—H16A	0.98
C5—C6	1.5269 (18)	C16—H16B	0.98
C5—C10	1.5524 (18)	C16—H16C	0.98
C6—C7	1.5260 (18)	C17—C18	1.3893 (19)
C6—H6A	0.99	C17—C22	1.3911 (18)
C6—H6B	0.99	C18—C19	1.383 (2)
C7—C8	1.524 (2)	C18—H18	0.95
C7—H7A	0.99	C19—C20	1.381 (2)
C7—H7B	0.99	C19—H19	0.95
C8—C9	1.5225 (19)	C20—C21	1.387 (2)
C8—H8A	0.99	C20—H20	0.95
C8—H8B	0.99	C21—C22	1.3845 (19)
C9—C10	1.5305 (18)	C21—H21	0.95
C9—H9A	0.99	C22—H22	0.95
C9—H9B	0.99		
			
C2—N1—O14	119.24 (10)	C10—C9—H9B	109.3
C2—N1—C5	116.41 (10)	H9A—C9—H9B	107.9
O14—N1—C5	117.39 (9)	C11—C10—C9	114.62 (11)
O13—C2—N1	125.63 (12)	C11—C10—C5	111.76 (11)
O13—C2—C3	127.65 (12)	C9—C10—C5	110.36 (10)
N1—C2—C3	106.72 (11)	C11—C10—H10	106.5
C2—C3—C4	105.52 (11)	C9—C10—H10	106.5
C2—C3—H3A	110.6	C5—C10—H10	106.5
C4—C3—H3A	110.6	C12—C11—C10	126.73 (13)
C2—C3—H3B	110.6	C12—C11—H11	116.6
C4—C3—H3B	110.6	C10—C11—H11	116.6
H3A—C3—H3B	108.8	C11—C12—H12A	120.0
C3—C4—C5	107.09 (10)	C11—C12—H12B	120.0
C3—C4—H4A	110.3	H12A—C12—H12B	120.0
C5—C4—H4A	110.3	N1—O14—C15	110.49 (9)
C3—C4—H4B	110.3	O14—C15—C17	110.98 (10)
C5—C4—H4B	110.3	O14—C15—C16	103.77 (10)
H4A—C4—H4B	108.6	C17—C15—C16	116.12 (11)
N1—C5—C6	110.47 (10)	O14—C15—H15	108.6
N1—C5—C4	99.85 (10)	C17—C15—H15	108.6
C6—C5—C4	112.75 (11)	C16—C15—H15	108.6
N1—C5—C10	110.47 (10)	C15—C16—H16A	109.5
C6—C5—C10	109.45 (10)	C15—C16—H16B	109.5
C4—C5—C10	113.53 (11)	H16A—C16—H16B	109.5
C7—C6—C5	111.89 (11)	C15—C16—H16C	109.5
C7—C6—H6A	109.2	H16A—C16—H16C	109.5
C5—C6—H6A	109.2	H16B—C16—H16C	109.5
C7—C6—H6B	109.2	C18—C17—C22	118.66 (12)
C5—C6—H6B	109.2	C18—C17—C15	118.77 (12)
H6A—C6—H6B	107.9	C22—C17—C15	122.54 (12)
C8—C7—C6	111.07 (11)	C19—C18—C17	120.93 (13)
C8—C7—H7A	109.4	C19—C18—H18	119.5
C6—C7—H7A	109.4	C17—C18—H18	119.5
C8—C7—H7B	109.4	C20—C19—C18	119.98 (13)
C6—C7—H7B	109.4	C20—C19—H19	120.0
H7A—C7—H7B	108.0	C18—C19—H19	120.0
C9—C8—C7	111.06 (11)	C19—C20—C21	119.80 (13)
C9—C8—H8A	109.4	C19—C20—H20	120.1
C7—C8—H8A	109.4	C21—C20—H20	120.1
C9—C8—H8B	109.4	C22—C21—C20	120.10 (13)
C7—C8—H8B	109.4	C22—C21—H21	119.9
H8A—C8—H8B	108.0	C20—C21—H21	119.9
C8—C9—C10	111.81 (11)	C21—C22—C17	120.53 (13)
C8—C9—H9A	109.3	C21—C22—H22	119.7
C10—C9—H9A	109.3	C17—C22—H22	119.7
C8—C9—H9B	109.3		
			
O14—N1—C2—O13	−14.0 (2)	N1—C5—C10—C11	−52.82 (13)
C5—N1—C2—O13	−164.04 (13)	C6—C5—C10—C11	−174.67 (10)
O14—N1—C2—C3	167.24 (10)	C4—C5—C10—C11	58.38 (14)
C5—N1—C2—C3	17.17 (15)	N1—C5—C10—C9	178.38 (10)
O13—C2—C3—C4	177.61 (13)	C6—C5—C10—C9	56.53 (13)
N1—C2—C3—C4	−3.62 (15)	C4—C5—C10—C9	−70.42 (13)
C2—C3—C4—C5	−9.48 (15)	C9—C10—C11—C12	10.9 (2)
C2—N1—C5—C6	−141.15 (11)	C5—C10—C11—C12	−115.63 (15)
O14—N1—C5—C6	68.22 (13)	C2—N1—O14—C15	75.55 (13)
C2—N1—C5—C4	−22.22 (14)	C5—N1—O14—C15	−134.67 (10)
O14—N1—C5—C4	−172.85 (10)	N1—O14—C15—C17	64.29 (12)
C2—N1—C5—C10	97.60 (13)	N1—O14—C15—C16	−170.29 (9)
O14—N1—C5—C10	−53.03 (13)	O14—C15—C17—C18	−95.21 (13)
C3—C4—C5—N1	17.59 (13)	C16—C15—C17—C18	146.62 (12)
C3—C4—C5—C6	134.83 (12)	O14—C15—C17—C22	82.70 (14)
C3—C4—C5—C10	−99.97 (13)	C16—C15—C17—C22	−35.46 (17)
N1—C5—C6—C7	−178.92 (11)	C22—C17—C18—C19	−0.54 (19)
C4—C5—C6—C7	70.32 (14)	C15—C17—C18—C19	177.46 (12)
C10—C5—C6—C7	−57.07 (14)	C17—C18—C19—C20	0.3 (2)
C5—C6—C7—C8	56.61 (15)	C18—C19—C20—C21	0.1 (2)
C6—C7—C8—C9	−54.81 (15)	C19—C20—C21—C22	−0.4 (2)
C7—C8—C9—C10	55.59 (15)	C20—C21—C22—C17	0.2 (2)
C8—C9—C10—C11	176.19 (11)	C18—C17—C22—C21	0.28 (19)
C8—C9—C10—C5	−56.57 (14)	C15—C17—C22—C21	−177.64 (12)

### Hydrogen-bond geometry (Å, º)

**Table:**

*D*—H···*A*	*D*—H	H···*A*	*D*···*A*	*D*—H···*A*
C15—H15···O13	1.00	2.42	3.0437 (16)	120
C18—H18···O13^i^	0.95	2.53	3.2864 (17)	136
C20—H20···O13^ii^	0.95	2.61	3.4307 (17)	145

Symmetry codes: (i) −*x*, −*y*, −*z*+1; (ii) *x*, *y*−1, *z*.
